# Untangling the relationship between diet and visceral fat mass through blood metabolomics and gut microbiome profiling

**DOI:** 10.1038/ijo.2017.70

**Published:** 2017-04-04

**Authors:** T Pallister, M A Jackson, T C Martin, C A Glastonbury, A Jennings, M Beaumont, R P Mohney, K S Small, A MacGregor, C J Steves, A Cassidy, T D Spector, C Menni, A M Valdes

**Affiliations:** 1 Department of Twin Research and Genetic Epidemiology, Kings College London, London, UK; 2 Department of Nutrition, Norwich Medical School, University of East Anglia, Norwich, UK; 3 Metabolon Inc., Durham, NC, USA; 4 Academic Rheumatology Clinical Sciences Building, University of Nottingham, Nottingham City Hospital, Nottingham, UK

## Abstract

**Background/Objectives:**

Higher visceral fat mass (VFM) is associated with an increased risk for developing cardio-metabolic diseases. The mechanisms by which an unhealthy diet pattern may influence visceral fat (VF) development has yet to be examined through cutting-edge multi-omic methods. Therefore, our objective was to examine the dietary influences on VFM and identify gut microbiome and metabolite profiles that link food intakes to VFM.

**Subjects/Methods:**

In 2218 twins with VFM, food intake and metabolomics data available we identified food intakes most strongly associated with VFM in 50% of the sample, then constructed and tested the ‘VFM diet score’ in the remainder of the sample. Using linear regression (adjusted for covariates, including body mass index and total fat mass), we investigated associations between the VFM diet score, the blood metabolomics profile and the fecal microbiome (*n*=889), and confirmed these associations with VFM. We replicated top findings in monozygotic (MZ) twins discordant (⩾1 s.d. apart) for VFM, matched for age, sex and the baseline genetic sequence.

**Results:**

Four metabolites were associated with the VFM diet score and VFM: hippurate, alpha-hydroxyisovalerate, bilirubin (Z,Z) and butyrylcarnitine. We replicated associations between VFM and the diet score (beta (s.e.): 0.281 (0.091); *P*=0.002), butyrylcarnitine (0.199 (0.087); *P*=0.023) and hippurate (−0.297 (0.095); *P*=0.002) in VFM-discordant MZ twins. We identified a single species, *Eubacterium dolichum* to be associated with the VFM diet score (0.042 (0.011), *P*=8.47 × 10^−5^), VFM (0.057 (0.019), *P*=2.73 × 10^−3^) and hippurate (−0.075 (0.032), *P*=0.021). Moreover, higher blood hippurate was associated with elevated adipose tissue expression neuroglobin, with roles in cellular oxygen homeostasis (0.016 (0.004), *P*=9.82x10^−6^).

**Conclusions:**

We linked a dietary VFM score and VFM to *E. dolichum* and four metabolites in the blood. In particular, the relationship between hippurate, a metabolite derived from microbial metabolism of dietary polyphenols, and reduced VFM, the microbiome and increased adipose tissue expression of neuroglobin provides potential mechanistic insight into the influence of diet on VFM.

## Introduction

Increased visceral fat (VF) is a strong risk factor for cardio-metabolic diseases. Observational studies have found that intakes of fruit,^1^ dairy^1^ and nutrients,^2^ and whole grains,^3^ and fiber^2, 4^ are protective, whereas intakes of fried foods and fat,^1, 5^ alcohol, red and processed meat^1^ and related nutrients,^2^ sugar-sweetened beverages^1, 5, 6, 7^ and refined grains^1, 3^ and high glycemic index foods^8, 9^ are associated with higher levels of VF mass (VFM) or waist circumference.

Over the past decade, studies on dietary patterns have emerged to examine the impact of the whole diet on metabolic health. In a recent study, authors created a protective diet score using self-reported intakes of favorable and unfavorable foods to investigate gene × diet interactions in obesity in 68 317 subjects of European ancestry.^10^ In another study of 48 631 European men and women, Angquist *et al.*^1^ created a summary score combining intakes of all food groups associated with changes in waist circumference over a median of 5.5 years. These large studies have confirmed the utility of this approach to studying VF interactions with diet, although the method has yet to be applied to omic data.

Metabolomics is being used to bridge the knowledge gap between diet and its effect on metabolic diseases. We recently showed that blood metabolites related to VFM link the impact of VFM on type 2 diabetes, insulin resistance and blood pressure.^11^ Moreover, we found reported food intakes to be associated with 106 different metabolites,^12^ establishing the central role of food intake on metabolic traits. However, the metabolomics profile of a metabolically unhealthy diet has not been thoroughly characterized and those metabolites linking diet to VFM development been distinguished.

Emerging evidence suggests a role for the intestinal microbiota in VF development by interacting with dietary components and contributing to the metabolomics profile.^13^ Early studies using rodents fed high-fat (HF) diets, have shown HF feeding to increase Firmicutes and decrease Bacteriodetes,^14^ reduce the class Clostridia,^15^ and increase sulfidogenic bacteria.^16^ Through modulating the gut microbiome profile, polyphenols from cranberry^17^ and pomegranate,^18^ resveratrol^18^ and gluco-oligosaccharide^19^ have shown to be protective of obesity in HF feeding.

The aims of this study were to identify foods most strongly associated with VFM in a population of UK twins, to develop and test a predictive dietary VFM-risk score using these food intakes, and to link the blood metabolomics and gut microbiome profiles of the score to VFM.

## Materials and methods

Twins enrolled in the TwinsUK registry, a register of UK adult twins,^20^ were included in the study. Twins were recruited throughout the UK primarily by media campaigns without selecting for specific diseases or traits. Food intakes were determined by a 131-item validated food frequency questionnaire^21^ between 1995 and 2001, in 2007 and 2014 to 2015. Quality control, subject exclusion criteria and methods for nutrient determination from food frequency questionnaire data have been described previously.^22^ Food frequencies were combined into 20 different food types before analysis (Supplementary Table S1). Other relevant phenotypic data include body mass index (BMI) and zygosity, which were determined by methods outlined previously.^20^ The study was approved by the St Thomas’ Hospital Research Ethics committee and all subjects provided informed written consent.

### Visceral fat mass

Visceral fat mass (VFM; g) was determined in 3457 female and 142 male twins by DXA (Dual-Energy X-ray Absorptiometry; Hologic QDR; Hologic, Inc., Waltham, MA, USA) whole-body scanning (supine) at a clinical visit by a trained research nurse. The QDR System Software Version 12.6 (Hologic, Inc., Waltham, MA, USA) was used to analyze the scans. VFM was calculated from one cross-section of the whole body at L4–L5, the typical location of a computed tomography slice. Subjects were excluded from the analysis if their VFM was 4 s.d. outside of the mean VFM. VFM did not follow a normal distribution and was normalized using a rank-based inverse normalization.

### Metabolomic profiling

Non-targeted mass spectroscopy-based metabolomic profiling was conducted by the metabolomics provider Metabolon, Inc. (Durham, NC, USA) on 6056 fasting blood samples, as described previously.^23, 24^ During the twin’s annual visit to St Thomas’ Hospital, fasted blood samples were collected by a trained research nurse and stored at −80 °C until further metabolomic processing. The Metabolon platform identified 292 structurally named biochemicals categorized into the following broad categories: amino acids, carbohydrates, vitamins, lipids, nucleotides, peptides and xenobiotics. Quality control on the metabolomics data set was performed as previously described.^23, 24^ Raw data were median normalized by dividing metabolite concentrations by the day median of that metabolite and then inverse normalized. For the metabolomics analysis, we included 2218 male (*n*=4) and female (*n*=2214) twins who had metabolomics profiling, BMI and VFM data available within and including ±5 years of food frequency questionnaire completion.

### Gut microbiome profiles

Fecal samples were collected at home by the twins and stored in the refrigerator for 2 days or less before their annual clinical visit at St Thomas’ Hospital. Samples were stored at −80 °C until further processing. Bacterial profiles were generated using 16S ribosomal RNA gene sequencing. Microbial DNA extracted, amplified, sequenced and processed, as part of a prior study^25^ (see reference for details), with an additional ∼1000 samples collected and processed under the same protocols. Sequencing reads were summarized as operational taxonomic units (OTUs) at 97% sequence similarity. This was carried out using UCLUST open-reference clustering against Greengenes v13_5 reference^26^ within QIIME 1.7.0,^27^ 6.2 % of the total sequences did not cluster to the reference and were excluded.^25^

OTUs that were observed in fewer than 25% of individuals were not considered for further study. Of 9840 OTUs (after removing singletons), 16% passed this threshold, resulting in a final set of 2118 OTUs. All OTU counts (including those in <25% of individuals) were collapsed into taxonomies at the family (124 variables), genus (283 variables) and species (153 variables) levels where only fully classified taxa were considered within each level. Alpha diversity was measured using Shannon’s phylogenetic diversity^28^ (also using QIIME) after rarefaction of the complete OTU table to 10 000 reads per sample. OTUs were adjusted for technical covariates including sequencing run and number of sequences in each sample using linear regression. The data were normalized using rank-based inverse normalization. For this study, we analyzed a subsample of the food frequency questionnaire, VFM and metabolomics sample for which we also had fecal microbiome profiling (*n*=889).

### Muther expression data

Gene expression of abdominal fat samples in 825 individuals were analyzed with the Illumina Human HT-12 V3 (Illumina, Inc., San Diego, CA, USA) for the Muther study, as described previously.^29^ In all, 586 individuals were analyzed for expression association with the top metabolite using a random intercept linear regression including age, BMI, metabolite batch, expression batch and family relatedness.

### Statistical analysis

Statistical analysis was carried out using Stata version 12 (StataCorp LLC, College Station, TX, USA).

**Figure 1:**
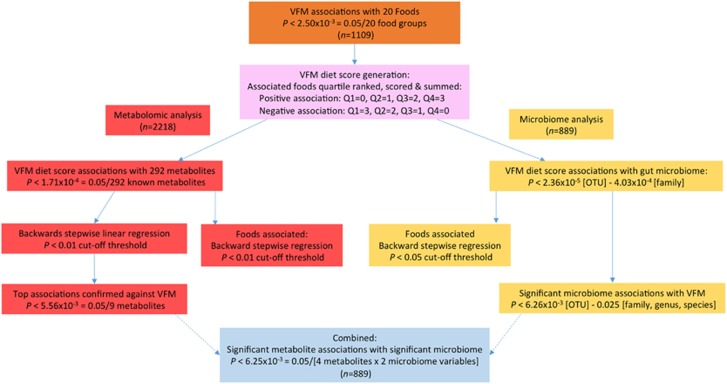
Outline of the study design.

Figure 1 summarizes the protocol and the specific details of data analysis are as follows:

### VFM food type associations and diet score formation and heritability

To determine significant associations between food intakes and VFM, we first randomly allocated twins to two groups (the training and test groups) ensuring co-twins assigned to the same group. In the training group (*n*=1109), a linear regression was performed for each of the 20 food groups (predictor variable) adjusted for covariates (total fat mass, age, sex, height^2^, family relatedness, dual-energy x-ray absorptiometry batch) with VFM (residual adjusted for BMI) as the response variable. Associations were considered significant if they passed the Bonferroni cutoff for multiple testing (*P*<2.50 × 10^−3^=(0.05/20 food groups)). Food groups significantly associated with VFM were included in the final score. To calculate the score, reported consumption frequencies of these food groups were quartile ranked and the quartiles assigned a score of 0–3 according to direction of the association (that is, positive association: Q1=0, Q2=1, Q3=2, Q4=3; negative association: Q1=3, Q2=2, Q3=1, Q4=0). Therefore, a higher VFM diet score is associated with a poorer diet quality. Following score assignment, scores for all variables were summed with the final score ranging from 0 to 15. Heritability of the VFM diet score was determined using linear structural equation modeling in Mx,^30, 31^ details can be found in Supplementary Text S1.

#### Binary classification test

In the test group (*n*=1109), the VFM diet score was first calculated as described above and then fitted into a logistic regression model adjusted for covariates (total fat mass, age, sex, height^2^, family relatedness, dual-energy x-ray absorptiometry batch and BMI category (1: <18.5 kg m^−2^; 2: ⩾18.5–24.9 kg m^−^^2^; 3: ⩾25–29.9 kg m^−^^2^; 4: ⩾30 kg m^−^^2^)) with the lower tertile of VFM assigned a negative outcome (0; *n*=369), and the top (high VFM) tertile of VFM considered a positive outcome (1; *n*=370). A binary classification test was then conducted to evaluate the predictive ability of the VFM diet score. The ability of the VFM diet score to correctly classify subjects with high VFM (sensitivity; true-positive rate) and correctly classify subjects with low VFM (specificity; true-negative rate) of the model was predicted and the receiver operating characteristic curve generated by plotting the true-positive rate against the false-positive rate at multiple threshold settings.

### VFM diet score and VFM associations with metabolomics and the microbiome

Details of the statistical analysis for the associations between the VFM diet score and VFM with blood metabolomics and microbiome taxa can be found in Supplementary Text S2.

## Results

### VFM food group associations

The characteristics of the study population can be found in Supplementary Table S3.

**Figure 2:**
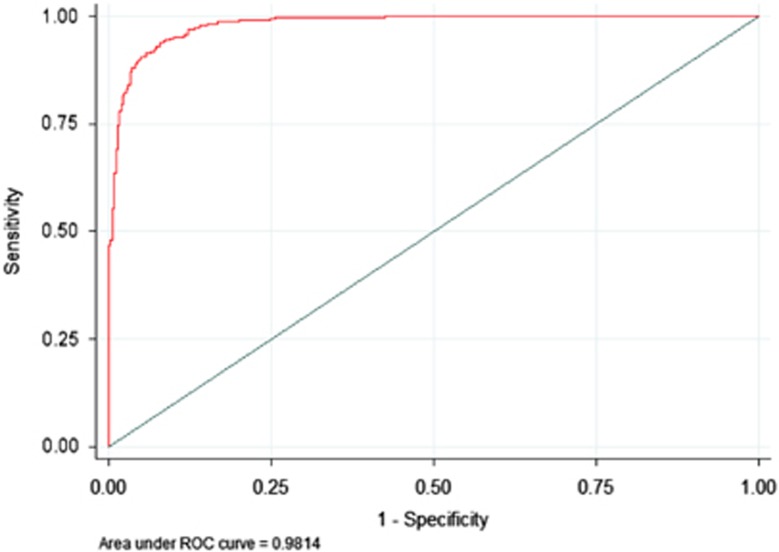
Receiver operating characteristic curve for the VFM diet score ability to predict the bottom and top tertiles of VFM.

We identified five significant food type associations with VFM in the training data set, including: fruits (−0.005 (0.001); *P*=1.95 × 10^−5^), red, processed meat and eggs (0.016 (0.005); *P*=3.94 × 10^−4^), fermented dairy products (−0.011 (0.004); *P*=1.14 × 10^−3^), fried and fast foods (0.015 (0.005); *P*=1.18 × 10^−3^) and whole-grain products (−0.008 (0.002); *P*=1.27 × 10^−3^). We next generated and evaluated the VFM diet score in the test group. The sensitivity of the VFM diet score was 93.72%, the specificity was 92.70% and overall 93.21% of subjects were classified into the correct VFM tertile. Figure 2 shows the receiver operating characteristic curve (area under the curve : 0.9841 (95% confidence interval (CI): 0.9772, 0.9911)). The association between the diet score and VFM was significant in VFM-discordant monozygotic (MZ) twins (0.281 (0.091); *P*=0.002). The diet score was strongly heritable (*h*^*2*^>40%) at 44% (95% CI: 37, 50%) (Supplementary Table S4).

**Figure 3:**
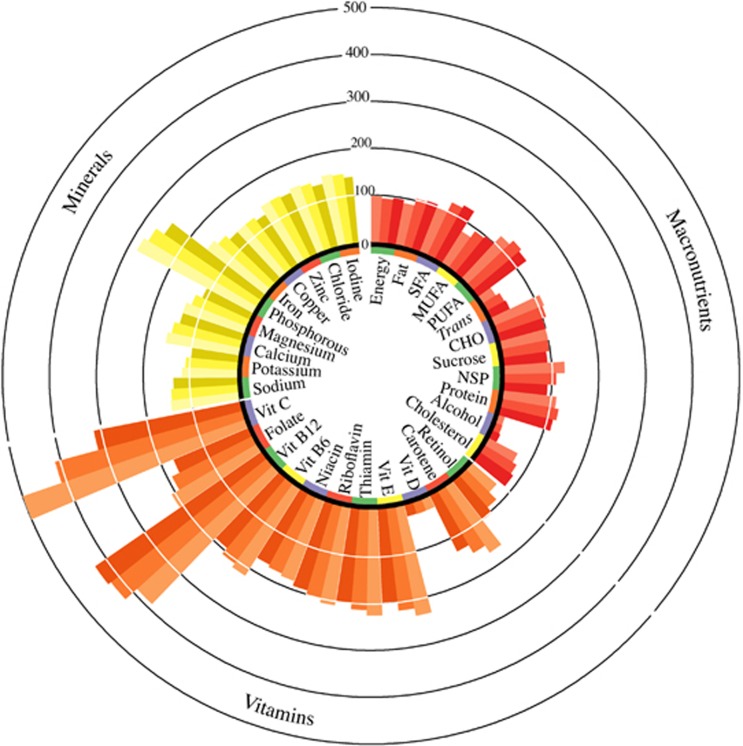
Nutrient profile of the VFM diet score presented as percentages of the UK dietary reference values by tertile of the VFM diet score. Average nutrient intakes by increasing tertile of the VFM diet score from clockwise (lightest to darkest) were assessed for percentage of the recommended intakes for 55-year-old women (according to the UK Dietary Reference Values).^56^ Using VFM diet score by tertile as the predictor of the residual energy adjusted nutrient intakes in a linear regression, statistically significant trends (*P*<0.001) were observed for all nutrients, except polyunsaturated fatty acids, protein, zinc and vitamin D. Carotene and retinol are represented as percentage of the recommended intake for total retinol equivalents. There is no UK DRV for vitamin D therefore 10 μg day^−1^ was used. CHO, carbohydrates; MUFAs, monounsaturated fatty acids; NSP, non-starch polysaccharides; PUFAs, polyunsaturated fatty acids; SFAs, saturated fatty acids; *Trans*, *trans* fatty acids; vit, vitamin.

The nutrient profile of the VFM diet score is shown in Figure 3 (Supplementary Table S5).

### VFM diet score metabolomics associations

We identified 30 metabolites significantly associated (*P*<1.71 × 10^−4^) with the VFM diet score after adjusting for covariates and multiple testing (Supplementary Table S6).

Following an adjustment for intakes of other foods (Supplementary Table S6), all associations between metabolites and the VFM diet score remained strong (*P*<0.01) although 6 no longer passed adjustment for multiple testing, suggesting intakes of other foods may be important for these metabolite associations.

### Metabolites associated with the VFM diet score and food groups independently

Eighteen metabolites were significantly (*P*<3.33 × 10^−4^ (0.05/(5 food groups × 30 metabolites))) associated with the food groups forming the VFM diet score following backward regression with all food groups (Supplementary Table S6). Notably, fruit intake was significantly associated with 11 metabolites. Whole-grain intake was significantly associated with five metabolites, red, processed meat, and eggs with three metabolites, and fried and fast food intakes with two metabolites.

### Metabolites associated with both the VFM diet and VFM

Following a backward stepwise linear regression including all 30 metabolites, 9 metabolites (accounting for 14% of the variance) remained significantly associated with the VFM diet score (Table 1). After adjusting for multiple testing (*P*<5.56 × 10^−3^), four of them were significantly associated with VFM independently of diet.

**Figure 4:**
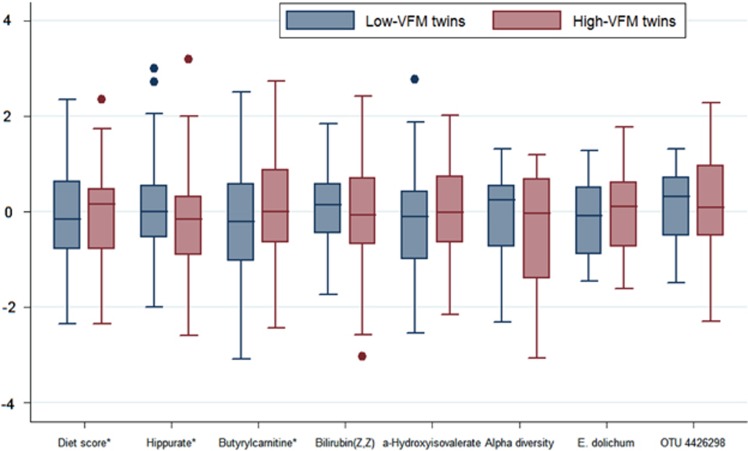
Comparisons of the VFM diet score, alpha diversity and top microbiome and metabolite associations in the low and high MZ VFM-discordant twins. All variables were standardized to have mean=0, s.d.=1. A linear regression was conducted using the VFM diet score, alpha diversity (Shannon index) and top microbiome and metabolite associations to predict VFM in the MZ-discordant (1 s.d. apart in VFM) twin sample. Significantly (*P*<0.05) higher VFM diet scores and butyrylcarnitine, and lower hippurate were observed with increasing VFM (*).

Reduced hippurate and bilirubin (Z,Z), and increased alpha-hydroxyisovalerate and butyrylcarnitine were all associated with increased VFM diet scores and VFM independently of the VFM diet and total body fat (Table 1). Associations between VFM and butyrylcarnitine (0.199 (0.087); *P*=0.023) and hippurate (−0.297 (0.095); *P*=0.002) were significant in VFM-discordant MZ twins (Figure 4; Supplementary Table S7). The metabolites explained on average 18.5% of the variance (range: 13.5–28.9%) in the association between the VFM diet score and VFM (Table 1).

### VFM diet score microbiome associations

Increased scores on the VFM diet were associated with reduced gut microbiome diversity (Shannon index; −0.025 (0.009), *P*=6.26 × 10^−3^), this association remained significant but was attenuated following adjustment for VFM (−0.020 (0.010), *P*=0.035).

Eight OTUs (Supplementary Table S8) and six taxa (Table 2) were significantly associated with the VFM diet score. The associations remained nominally significant (*P*<0.05) following an adjustment for intakes of other foods (Table 2).

### Microbiome taxa associated with both the VFM diet and VFM

Increased abundance of the species *Eubacterium dolichum* (0.057 (0.019), *P*=2.73 × 10^−3^) was significantly associated with higher VFM and a *Bifidobacterium* OTU (OTU ID: 4426298; −0.046 (0.016), *P*=0.005) with lower VFM, both independently of the VFM diet score. We found that 16.4% of the effect of the VFM diet score on VFM (r^2^_x_=0.0238) was mediated by *E. dolichum* (r^2^_xy_= 0.0199) and 17.2% by the *Bifidobacterium* OTU.

### *E. dolichum* and hippurate associated with both VFM and VFM diet

We tested associations with those four metabolites associated with both VFM and the VFM diet for their association with *E. dolichum* and the *Bifidobacterium* OTU. We identified increased abundances of *E. dolichum* to be associated with significantly lower levels of hippurate at the nominal level (*P*<0.05) independently of VFM, the VFM diet score, Shannon index and covariates (−0.075 (0.032), *P*=0.021). We further determined that 36.9% of the effect of *E. dolichum* on VFM (r^2^_x_= 0.0065) was mediated by hippurate (r^2^_xy_=0.0041) after adjusting for diet and covariates.

### Hippurate association with adipose tissue transcriptome

We found increased levels of hippurate neuroglobin in the greater twin population to be associated with elevated adipose tissue expression of neuroglobin, a member of the vertebrate globin family involved in cellular oxygen homeostasis (0.016 (0.004), *P*=9.82 × 10^−6^).

## Discussion

In this study, using a newly developed dietary VFM-risk score, authenticated in the test population, we have characterized for the first time the blood metabolomics profile of a dietary pattern predictive of VFM and have identified a specific gut bacterial species associated with this pattern and VFM after adjusting for a range of confounders. Our novel data have highlighted the species *E. dolichum* in the gut and hippurate in blood may link diet to VFM.

Our score was highly predictive of VFM in our population (including in MZ twins discordant for VFM), which allowed us to investigate the impact of diet on VFM development using metabolomics and microbiome methods. Four metabolites were associated with both the VFM diet score and VFM (independently of diet). They included reduced hippurate and bilirubin (Z,Z), and increased alpha-hydroxyisovalerate and butyrylcarnitine with increasing VFM diet scores and VFM. Alpha-hydroxyisovalerate and butyrylcarnitine are metabolites of branched-chain amino acid catabolism and fatty acid metabolism that have been found to be elevated in obese children^32^ and adults.^33^ Moreover, alpha-hydroxyisovalerate has been identified as an important predictor of insulin resistance and glucose intolerance.^34, 35^ Both metabolites were associated with higher intakes of red and processed meats and eggs. Animal derived fats and protein have not been specifically linked to disrupted branched-chain amino acid metabolism in humans although under HF feeding in mice the addition of branched-chain amino acid exacerbates insulin resistance through stimulating the mTOR kinase pathway.^36^

Bilirubin is involved in hemoglobin and prophyrin metabolism and also acts as an endogenous anti-oxidant. Reflecting our findings, lower levels of serum bilirubin have been found to correlate with increased abdominal adiposity and metabolic complications.^37, 38, 39^ Higher intakes of fried and fast foods were significantly associated with lower bilirubin (Z,Z). Higher intakes of total fatty acids have previously been associated with lower serum bilirubin,^39^ which may be related to increased oxidative stress depleting bilirubin levels. Vegetable oil frying reduces oil polyphenols and when fed to mice increases liver microsomal lipid peroxides.^40^

Hippurate appeared to be the most important metabolite linking diet to VFM. Hippurate is a mammalian–microbial co-metabolite, which is a glycine conjugate of benzoic acid formed in the mitochondria of the liver^41^ and kidneys,^42^ as well as through gut bacterial production of benzoic acid from dietary components, primarily polyphenols.^43, 44^ Similarly, we found hippurate to be associated with increased intakes of fruit and whole-grain products. Studies, which measured urinary or serum levels of hippurate in the context of obesity or metabolic diseases, have mainly been limited to animal models, which have shown reduced urinary hippuric acid excretion in obesity^45, 46, 47^ and elevated levels in type 2 diabetes^48^ compared with controls. We found increased hippurate in blood to be associated with elevated adipose tissue expression of neuroglobin, a type of globin primarily expressed in neurons and some endocrine tissues,^49^ which protects cells against hypoxia and oxidative stress.^50^ Neurglobin expression in adipose tissue has not been studied extensively. Although the process of hypoxia has recently emerged within the literature as a potential mechanism in the development of adipose tissue dysfunction.^51^ This highlights a potential means by which hippurate may protect against adipose tissue dysfunction and VFM development as a result.

The species *E. dolichum* within the family *Erysipelotrichaceae* was positively associated with the dietary VFM score and VFM, suggesting a role of this microbe in VFM development modulated by diet (in particular whole-grain consumption). In a mouse model of Western-style diet induced obesity, *E. dolichum* was found to be elevated,^52^ moreover metagenomics analysis demonstrated the *E. dolichum* genome to be enriched for phosphotransferase proteins with functions in the import and processing of simple sugars. In another study of two Japanese quail strains (atherosclerotic-resistant and non-resistant), *E. dolichum* was overabundant when atherosclerotic-resistant quails were fed a high cholesterol diet compared with control.^53^ We believe we are the first to identify a link between this species and a HF, low-fiber diet in human subjects although no literature exists as to the metabolic implications of this species. It is possible the association between *E. dolichum* and VFM may primarily be an artifact of poor diet rather than a factor contributing to VFM, although the association did remain significant when adjusting for the VFM dietary risk score. The relationship between *E. dolichum* and hippurate in our data set is likely complex and it is beyond the capacity of our data set to be adequately explored.

Our study had a number of strengths. We believe we are the first large-scale study to use multi-omic methods to investigate the impact of diet on VFM. The dietary components of our VFM score replicate findings from previous epidemiological studies^1, 3, 5^ justifying the strength/validity of our score. Like VFM,^54^ we found this score to be strongly determined by genetics (*h*^*2*^: 44%), which agrees with previous findings where the heritability of ‘unhealthy’ diet patterns ranged from 33 to 50%.^55^ The limitations of our study also warrant discussion. First, our population was predominantly female and therefore, our results may not apply to men. As our study is cross-sectional, it does not allow us to attribute cause and effect to our findings. Although we adjusted for possible confounders there is still the possibility of residual or unmeasured confounding from additional unmeasured factors or measurement error. However, given our detailed adjustment for a comprehensive set of confounders and adjustment for multiple testing it is unlikely that these would account fully for the observed results. Our characterization of the gut microbiome was also limited by the use of 16S gene sequencing. Further investigation using metagenomic approaches may provide a deeper understanding of the microbe–metabolite interactions at a functional level. Different time points were used for different samples, although likely our results would improve if the same time point was used. We did not have repeated measurements for subjects and could therefore not examine intra-individual variation. We did not replicate our findings in an independent population, although we were able to replicate the associations between VFM and the diet score, hippurate and butyrlcarnitine in MZ twins discordant for VFM, who are matched for age, gender and the baseline genetic sequence.

## Conclusions

An unhealthy dietary pattern is a strong determinant of VFM. Using this unique data set, we linked a dietary VFM score and VFM to a gut microbial species and metabolites in the blood. Specifically, in our population the species *E. dolichum* appears to link the intake of a diet low in fruit, whole grains and fermented dairy products and high in red, processed meat and eggs and fried and fast foods to VFM. Moreover, we identified hippurate, a microbial metabolite involved in benzoate metabolism, to link these components to the microbiome. Hippurate was in turn associated with adipose expression of neuroglobin, suggesting a plausible mechanism of interaction. Future studies should aim to confirm these results in a dietary intervention setting and explore the health implications of our findings.

## Figures and Tables

**Table 1:** List of metabolites independently associated with the VFM diet score (*P*<0.01 in backward linear regression), their association with VFM and the proportion of the association of the VFM diet score with VFM that is mediated by the VFM diet score association with the metabolites (*P*<5.56 × 10^−3^)

*Metabolite name*	*VFM diet score stepwise*^a^		*VFM*^b^		*Diet R*^*2*^ *no metabolite*^c^	*Diet R*^*2*^ *with metabolite*^d^	*% Association through metabolite*
	*Beta (s.e.)*	P*-value*	*Beta (s.e.)*	P*-value*			
Hippurate	−0.45 (0.10)	2.15 × 10^−5^	-0.081(0.012)	1.33 × 10^−11^	0.0312	0.0222	28.8%
Alpha-hydroxyisovalerate	0.38 (0.10)	9.60 × 10^−5^	0.050 (0.013)	1.65 × 10^−4^		0.0270	13.5%
Butyrylcarnitine	0.33 (0.10)	8.54 × 10^−4^	0.072 (0.013)	5.86 × 10^−8^		0.0267	14.4%
Bilirubin (Z,Z)	−0.31 (0.10)	1.76 × 10^−3^	−0.049 (0.013)	1.88 × 10^−4^		0.0258	17.3%
Indolepropionate	−0.33 (0.11)	2.21 × 10^−3^	−0.030 (0.012)	1.40 × 10^−2^			
1-Arachidonoylglycerophosphocholine*	0.27 (0.10)	5.20 × 10^−3^	0.031 (0.012)	1.07 × 10^−2^			
EPA (20:5n3)	−0.75 (0.10)	1.13 × 10^−13^	0.020 (0.012)	NS			
Threonate	−0.32 (0.11)	2.59 × 10^−3^	−0.016 (0.012)	NS			
X-11793—Oxidized bilirubin*	0.33 (0.11)	2.63 × 10^−3^	−0.004 (0.012)	NS			

Abbreviations: BMI, body mass index; EPA, eicosapentaenoate; NS, not significant; VFM, visceral fat mass.

NS: *P*>0.05.

The asterisk (*) on the end of the metabolite name indicates the metabolite identity has not been confirmed by accurate mass data. The proportion of the variance in VFM explained by the VFM diet score after taking into account all covariates as in (^a^) and adjusting for the metabolite.

a Thirty metabolites significantly associated with the VFM diet score (Table 2) were adjusted for covariates (batch effects, age, BMI and sex) and fitted into a backward stepwise linear regression to predict the VFM diet score using *P*<0.01 as the threshold cutoff.

b Nine metabolites independently associated with the VFM diet score were tested for their association with VFM adjusted for covariates (age, batch effects, BMI, total fat, sex, height^2^ and family relatedness). Associations passing the Bonferonni cutoff were considered significant (*P*<5.56x10^−3^).

c The proportion of the variance in VFM explained by the VFM diet score after taking into account all covariates (age, sex, BMI, height^2^ and batch effects).

d The proportion of the variance in VFM explained by the VFM diet score after taking into account all covariates as in (c) plus the metabolite.

**Table 2:** List of taxa associated with the VFM diet score (unadjusted and adjusted for other food intakes), their association with foods forming the VFM diet score and their independent association with the VFM diet score (*P*<0.05 in backward linear regression)

*Taxon*	*Level*	*VFM score*^a^		*VFM score adjusted foods*^b^		*Foods associated*^c^
		*Beta (s.e.)*	P*-value*	*Beta (s.e.)*	P*-value*	P*<0.05*
*Actinomyces*	Genus	0.052 (0.011)	9.77 × 10^−7^	0.052 (0.011)	9.77 × 10^−7^	FF (0.028 (0.009)^d^ RM (0.027 (0.008))^d^
*Lachnospira*	Genus	−0.045 (0.009)	2.79 × 10^−6^	−0.038 (0.010)	8.33 × 10^−5^	Fruit (0.006 (0.002))
Actinomycetaceae	Family	0.043 (0.011)	5.47 × 10^−5^	0.043 (0.011)	5.47 × 10^−5^	FF (0.021 (0.010)) RM (0.024 (0.008))
*Eubacterium dolichum^e^*	Species	0.042 (0.011)	8.47 × 10^−5^	0.043 (0.011)	6.19 × 10^−5^	WG (−0.010 (0.004))
*Veillonella dispar*	Species	−0.039 (0.011)	3.05 × 10^−4^	−0.031 (0.011)	4.00 × 10^−3^	None
*Anaeroplasmataceae*	Family	−0.037 (0.010)	3.75 × 10^−4^	−0.036 (0.010)	3.37 × 10^−4^	Fruit (0.007 (0.003)) WG (0.011 (0.004))

Abbreviations: BMI, body mass index; FF, fried and fast foods; RM, red meat; VFM, visceral fat mass; WG

, whole-grain products.

a Taxa associations with the VFM diet score were adjusted for covariates (age, Shannon index, BMI and sex) and multiple testing.

b The VFM diet score and 15 food groups not forming the score were fitted into a backward stepwise linear regression model to predict each significant taxon using *P*<0.05 as the cutoff threshold.

c All 20 food groups were fitted into a backward stepwise linear regression model to predict each significant taxon using *P*<0.05 as the cutoff threshold. Significant results shown only for foods forming the VFM diet score.

d Statistically significant: *P*<0.0025.

e *E. dolichum* is the only taxon associated with VFM independently of the VFM diet score (beta (s.e.): 0.057 (0.019), *P*=2.74 × 10^–3^).

## References

[bib1] Romaguera D, Angquist L, Du H, Jakobsen MU, Forouhi NG, Halkjaer J et al. Food composition of the diet in relation to changes in waist circumference adjusted for body mass index. PloS One 2011; 6: e23384. 21858094 10.1371/journal.pone.0023384 PMC3157378

[bib2] Fischer K, Moewes D, Koch M, Muller HP, Jacobs G, Kassubek J et al. MRI-determined total volumes of visceral and subcutaneous abdominal and trunk adipose tissue are differentially and sex-dependently associated with patterns of estimated usual nutrient intake in a northern German population. Am J Clin Nutr 2015; 101: 794–807. 25833977 10.3945/ajcn.114.101626

[bib3] Caron-Jobin M, Morisset AS, Tremblay A, Huot C, Legare D, Tchernof A. Elevated serum 25(OH)D concentrations, vitamin D, and calcium intakes are associated with reduced adipocyte size in women. Obesity (Silver Spring, MD) 2011; 19: 1335–1341. 10.1038/oby.2011.90 21527900

[bib4] Hairston KG, Vitolins MZ, Norris JM, Anderson AM, Hanley AJ, Wagenknecht LE. Lifestyle factors and 5-year abdominal fat accumulation in a minority cohort: the IRAS Family Study. Obesity (Silver Spring, MD) 2012; 20: 421–427. 10.1038/oby.2011.171 PMC3856431 21681224

[bib5] Mollard RC, Senechal M, MacIntosh AC, Hay J, Wicklow BA, Wittmeier KD et al. Dietary determinants of hepatic steatosis and visceral adiposity in overweight and obese youth at risk of type 2 diabetes. Am J Clin Nutr 2014; 99: 804–812. 24522441 10.3945/ajcn.113.079277

[bib6] Ma J, Sloan M, Fox CS, Hoffmann U, Smith CE, Saltzman E et al. Sugar-sweetened beverage consumption is associated with abdominal fat partitioning in healthy adults. J Nutr 2014; 144: 1283–1290. 24944282 10.3945/jn.113.188599 PMC4093984

[bib7] Odegaard AO, Choh AC, Czerwinski SA, Towne B, Demerath EW. Sugar-sweetened and diet beverages in relation to visceral adipose tissue. Obesity (Silver Spring, MD) 2012; 20: 689–691. 10.1038/oby.2011.277 PMC3288354 21901024

[bib8] Dal Molin Netto B, Landi Masquio DC, Da Silveira Campos RM, De Lima Sanches P, Campos Corgosinho F, Tock L et al. The high glycemic index diet was an independent predictor to explain changes in agouti-related protein in obese adolescents. Nutricion Hospitalaria 2014; 29: 305–314. 24528346 10.3305/nh.2014.29.2.7087

[bib9] Romaguera D, Angquist L, Du H, Jakobsen MU, Forouhi NG, Halkjaer J et al. Dietary determinants of changes in waist circumference adjusted for body mass index - a proxy measure of visceral adiposity. PloS One 2010; 5: e11588. 20644647 10.1371/journal.pone.0011588 PMC2904387

[bib10] Nettleton JA, Follis JL, Ngwa JS, Smith CE, Ahmad S, Tanaka T et al. Gene x dietary pattern interactions in obesity: analysis of up to 68 317 adults of European ancestry. Hum Mol Genet 2015; 24: 4728–4738. 25994509 10.1093/hmg/ddv186 PMC4512626

[bib11] Menni C, Migaud M, Glastonbury CA, Beaumont M, Nikolaou A, Small KS et al. Metabolomic profiling to dissect the role of visceral fat in cardiometabolic health. Obesity (Silver Spring, MD) 2016; 24: 1380–1388. 10.1002/oby.21488 PMC4914926 27129722

[bib12] Pallister T, Jennings A, Mohney RP, Yarand D, Mangino M, Cassidy A et al. Characterizing blood metabolomics profiles associated with self-reported food intakes in female twins. PloS One 2016; 11: e0158568. 27355821 10.1371/journal.pone.0158568 PMC4927065

[bib13] Shoaie S, Ghaffari P, Kovatcheva-Datchary P, Mardinoglu A, Sen P, Pujos-Guillot E et al. Quantifying diet-induced metabolic changes of the human gut microbiome. Cell Metab 2015; 22: 320–331. 26244934 10.1016/j.cmet.2015.07.001

[bib14] Taira R, Yamaguchi S, Shimizu K, Nakamura K, Ayabe T, Taira T. Bacterial cell wall components regulate adipokine secretion from visceral adipocytes. J Clin Biochem Nutr 2015; 56: 149–154. 25759521 10.3164/jcbn.14-74 PMC4345181

[bib15] Etxeberria U, Arias N, Boque N, Macarulla MT, Portillo MP, Milagro FI et al. Shifts in microbiota species and fermentation products in a dietary model enriched in fat and sucrose. Benef Microbes 2015; 6: 97–111. 25213025 10.3920/BM2013.0097

[bib16] Shen W, Wolf PG, Carbonero F, Zhong W, Reid T, Gaskins HR et al. Intestinal and systemic inflammatory responses are positively associated with sulfidogenic bacteria abundance in high-fat-fed male C57BL/6J mice. J Nutr 2014; 144: 1181–1187. 24919690 10.3945/jn.114.194332

[bib17] Anhe FF, Roy D, Pilon G, Dudonne S, Matamoros S, Varin TV et al. A polyphenol-rich cranberry extract protects from diet-induced obesity, insulin resistance and intestinal inflammation in association with increased Akkermansia spp. population in the gut microbiota of mice. Gut 2015; 64: 872–883. 25080446 10.1136/gutjnl-2014-307142

[bib18] Neyrinck AM, Van Hee VF, Bindels LB, De Backer F, Cani PD, Delzenne NM. Polyphenol-rich extract of pomegranate peel alleviates tissue inflammation and hypercholesterolaemia in high-fat diet-induced obese mice: potential implication of the gut microbiota. Br J Nutr 2013; 109: 802–809. 22676910 10.1017/S0007114512002206

[bib19] Serino M, Luche E, Gres S, Baylac A, Berge M, Cenac C et al. Metabolic adaptation to a high-fat diet is associated with a change in the gut microbiota. Gut 2012; 61: 543–553. 22110050 10.1136/gutjnl-2011-301012 PMC3292714

[bib20] Moayyeri A, Hammond CJ, Hart DJ, Spector TD. The UK adult twin registry (TwinsUK Resource). Twin Res Hum Genet 2013; 16: 144–149. 23088889 10.1017/thg.2012.89 PMC3927054

[bib21] Bingham SA, Welch AA, McTaggart A, Mulligan AA, Runswick SA, Luben R et al. Nutritional methods in the European Prospective Investigation of Cancer in Norfolk. Public Health Nutr 2001; 4: 847–858. 11415493 10.1079/phn2000102

[bib22] Teucher B, Skinner J, Skidmore PM, Cassidy A, Fairweather-Tait SJ, Hooper L et al. Dietary patterns and heritability of food choice in a UK female twin cohort. Twin Res Hum Genet 2007; 10: 734–748. 17903115 10.1375/twin.10.5.734

[bib23] Menni C, Kastenmuller G, Petersen AK, Bell JT, Psatha M, Tsai PC et al. Metabolomic markers reveal novel pathways of ageing and early development in human populations. Int J Epidemiol 2013; 42: 1111–1119. 23838602 10.1093/ije/dyt094 PMC3781000

[bib24] Menni C, Fauman E, Erte I, Perry JR, Kastenmuller G, Shin SY et al. Biomarkers for type 2 diabetes and impaired fasting glucose using a nontargeted metabolomics approach. Diabetes 2013; 62: 4270–4276. 23884885 10.2337/db13-0570 PMC3837024

[bib25] Goodrich Julia K, Waters Jillian L, Poole Angela C, Sutter Jessica L, Koren O, Blekhman R et al. Human genetics shape the gut microbiome. Cell 2014; 159: 789–799. 25417156 10.1016/j.cell.2014.09.053 PMC4255478

[bib26] DeSantis TZ, Hugenholtz P, Larsen N, Rojas M, LarsenBrodie EL, Keller K et al. Greengenes, a chimera-checked 16S rRNA gene database and workbench compatible with ARB. Appl Environ Microbiol 2006; 72: 5069–5072. 16820507 10.1128/AEM.03006-05 PMC1489311

[bib27] Caporaso JG, Kuczynski J, Stombaugh J, Bittinger K, Bushman FD, Costello EK et al. QIIME allows analysis of high-throughput community sequencing data. Nat Methods 2010; 7: 335–336. 20383131 10.1038/nmeth.f.303 PMC3156573

[bib28] Faith DP. Conservation evaluation and phylogenetic diversity. Biol Cons 1992; 61: 1–10.

[bib29] Grundberg E, Small KS, Hedman AK, Nica AC, Buil A, Keildson S et al. Mapping cis- and trans-regulatory effects across multiple tissues in twins. Nat Genet 2012; 44: 1084–1089. 22941192 10.1038/ng.2394 PMC3784328

[bib30] Neale MC, Cardon LR, Organization NATMethodology for Genetic Studies of Twins and Families vol. 67. Kluwer Academic Publishers: Dordrecht, The Netherlands, 1992.

[bib31] Neale MC, Boker SM, Xie G, Maes H. Mx: Statistical Modeling. Department of Psychiatry, Medical College of Virginia: Richmond, VA, USA, 2003.

[bib32] Butte NF, Liu Y, Zakeri IF, Mohney RP, Mehta N, Voruganti VS et al. Global metabolomic profiling targeting childhood obesity in the Hispanic population. Am J Clin Nutr 2015; 102: 256–267. 26085512 10.3945/ajcn.115.111872 PMC4515872

[bib33] Moore SC, Matthews CE, Sampson JN, Stolzenberg-Solomon RZ, Zheng W, Cai Q et al. Human metabolic correlates of body mass index. Metabolomics 2014; 10: 259–269. 25254000 10.1007/s11306-013-0574-1 PMC4169991

[bib34] Gall WE, Beebe K, Lawton KA, Adam KP, Mitchell MW, Nakhle PJ et al. alpha-hydroxybutyrate is an early biomarker of insulin resistance and glucose intolerance in a nondiabetic population. PloS One 2010; 5: e10883. 20526369 10.1371/journal.pone.0010883 PMC2878333

[bib35] Varvel SA, Pottala JV, Thiselton DL, Caffrey R, Dall T, Sasinowski M et al. Serum alpha-hydroxybutyrate (alpha-HB) predicts elevated 1 h glucose levels and early-phase beta-cell dysfunction during OGTT. BMJ Open Diabetes Res Care 2014; 2: e000038. 10.1136/bmjdrc-2014-000038 PMC4212560 25452875

[bib36] Newgard CB, An J, Bain JR, Muehlbauer MJ, Stevens RD, Lien LF et al. A branched-chain amino acid-related metabolic signature that differentiates obese and lean humans and contributes to insulin resistance. Cell Metab 2009; 9: 311–326. 19356713 10.1016/j.cmet.2009.02.002 PMC3640280

[bib37] Wu Y, Li M, Xu M, Bi Y, Li X, Chen Y et al. Low serum total bilirubin concentrations are associated with increased prevalence of metabolic syndrome in Chinese. J Diabetes 2011; 3: 217–224. 21631904 10.1111/j.1753-0407.2011.00138.x

[bib38] Kwon KM, Kam JH, Kim MY, Kim MY, Chung CH, Kim JK et al. Inverse association between total bilirubin and metabolic syndrome in rural Korean women. J Women's Health 2011; 20: 963–969. 10.1089/jwh.2010.2453 21671781

[bib39] Jenko-Praznikar Z, Petelin A, Jurdana M, Ziberna L. Serum bilirubin levels are lower in overweight asymptomatic middle-aged adults: an early indicator of metabolic syndrome? Metab Clin Exp 2013; 62: 976–985. 23414908 10.1016/j.metabol.2013.01.011

[bib40] Quiles JL, Huertas JR, Battino M, Ramirez-Tortosa MC, Cassinello M, Mataix J et al. The intake of fried virgin olive or sunflower oils differentially induces oxidative stress in rat liver microsomes. Br J Nutr 2002; 88: 57–65. 12117428 10.1079/BJNBJN2002588

[bib41] Gatley SJ, Sherratt HS. The synthesis of hippurate from benzoate and glycine by rat liver mitochondria. Submitochondrial localization and kinetics. Biochem J 1977; 166: 39–47. 901416 10.1042/bj1660039 PMC1164954

[bib42] Temellini A, Mogavero S, Giulianotti PC, Pietrabissa A, Mosca F, Pacifici GM. Conjugation of benzoic acid with glycine in human liver and kidney: a study on the interindividual variability. Xenobiotica 1993; 23: 1427–1433. 8135043 10.3109/00498259309059451

[bib43] Gonthier MP, Verny MA, Besson C, Remesy C, Scalbert A. Chlorogenic acid bioavailability largely depends on its metabolism by the gut microflora in rats. J Nutr 2003; 133: 1853–1859. 12771329 10.1093/jn/133.6.1853

[bib44] Walsh MC, Brennan L, Pujos-Guillot E, Sebedio JL, Scalbert A, Fagan A et al. Influence of acute phytochemical intake on human urinary metabolomic profiles. Am J Clin Nutr 2007; 86: 1687–1693. 18065587 10.1093/ajcn/86.5.1687

[bib45] Shearer J, Duggan G, Weljie A, Hittel DS, Wasserman DH, Vogel HJ. Metabolomic profiling of dietary-induced insulin resistance in the high fat-fed C57BL/6J mouse. Diabetes Obes Metab 2008; 10: 950–958. 18215169 10.1111/j.1463-1326.2007.00837.x PMC6996141

[bib46] Waldram A, Holmes E, Wang Y, Rantalainen M, Wilson ID, Tuohy KM et al. Top-down systems biology modeling of host metabotype-microbiome associations in obese rodents. J Proteome Res 2009; 8: 2361–2375. 19275195 10.1021/pr8009885

[bib47] Calvani R, Miccheli A, Capuani G, Tomassini Miccheli A, Puccetti C, Delfini M et al. Gut microbiome-derived metabolites characterize a peculiar obese urinary metabotype. Int J Obes 2010; 34: 1095–1098. 10.1038/ijo.2010.44 20212498

[bib48] Williams RE, Lenz EM, Evans JA, Wilson ID, Granger JH, Plumb RS et al. A combined (1)H NMR and HPLC-MS-based metabonomic study of urine from obese (fa/fa) Zucker and normal Wistar-derived rats. J Pharm Biomed Anal 2005; 38: 465–471. 15925248 10.1016/j.jpba.2005.01.013

[bib49] Burmester T, Weich B, Reinhardt S, Hankeln T. A vertebrate globin expressed in the brain. Nature 2000; 407: 520–523. 11029004 10.1038/35035093

[bib50] Burmester T, Gerlach F, Hankeln T. Regulation and role of neuroglobin and cytoglobin under hypoxia. Adv Exp Med Biol 2007; 618: 169–180. 18269196 10.1007/978-0-387-75434-5_13

[bib51] Kim JH, Kim SH, Song SY, Kim WS, Song SU, Yi T et al. Hypoxia induces adipocyte differentiation of adipose-derived stem cells by triggering reactive oxygen species generation. Cell Biol Int 2014; 38: 32–40. 23956071 10.1002/cbin.10170

[bib52] Turnbaugh PJ, Backhed F, Fulton L, Gordon JI. Diet-induced obesity is linked to marked but reversible alterations in the mouse distal gut microbiome. Cell Host Microbe 2008; 3: 213–223. 18407065 10.1016/j.chom.2008.02.015 PMC3687783

[bib53] Liu S, Bennett DC, Tun HM, Kim JE, Cheng KM, Zhang H et al. The effect of diet and host genotype on ceca microbiota of Japanese quail fed a cholesterol enriched diet. Front Microbiol 2015; 6: 1092. 26500632 10.3389/fmicb.2015.01092 PMC4595795

[bib54] Direk K, Cecelja M, Astle W, Chowienczyk P, Spector TD, Falchi M et al. The relationship between DXA-based and anthropometric measures of visceral fat and morbidity in women. BMC Cardiovasc Disord 2013; 13: 25. 23552273 10.1186/1471-2261-13-25 PMC3769144

[bib55] Pallister T, Spector TD, Menni C. Twin studies advance the understanding of gene-environment interplay in human nutrigenomics. Nutr Res Rev 2014; 27: 242–251. 25522675 10.1017/S095442241400016X

[bib56] Health. DoDietary reference values for food energy and nutrients for the United Kingdom. Report of the panel on dietary reference values of the Committee on Medical Aspects of Food Policy. Report on Health and Social Subjects 41. HMSO: London, 1991. 1961974

