# Supplementary material for: Untangling the relationship between diet and visceral fat mass through blood metabolomics and gut microbiome profiling
Source: Int J Obes (Lond). 2017 Apr 4;41(7):1106–13. doi: 10.1038/ijo.2017.70 (PMC5504448; doi:10.1038/ijo.2017.70)
Supplement: Supplementary Material [file ijo201770x1.docx]

**Supplementary Tables and Text**

**Untangling the Relationship Between Diet and Visceral Fat Mass Through Blood Metabolomics and Gut Microbiome Profiling**

Tess Pallister, Matthew A Jackson, Tiphaine C Martin, Craig A Glastonbury, Amy Jennings, Michelle Beaumont, Robert P Mohney, Kerrin S Small, Alexander MacGregor, Claire J Steves, Aedin Cassidy, Tim D Spector, Cristina Menni, Ana M Valdes

**Supplementary Table S1: Food items included in food groups**

| **Food group** | **FFQ items** |
| --- | --- |
| **Vegetables** | |
|  | Broccoli, spring green, kale |
|  | Brussel sprouts |
|  | Cabbage |
|  | Cauliflower |
|  | Coleslaw |
|  | Avocado |
|  | Beetroot |
|  | Marrow, courgettes |
|  | Mushrooms |
|  | Parsnips, turnips, swedes |
|  | Sweetcorn |
|  | Sweet peppers |
|  | Watercress |
|  | Carrots |
|  | Tomatoes |
|  | Garlic (clove) |
|  | Leeks |
|  | Onions |
|  | Green salad, lettuce, cucumber, celery |
|  | Spinach |
|  | Watercress |
|  | Vegetable soups (bowl) |
|  | Boiled, mashed, instant or one jacket potato |
| **Fruit** | |
|  | Strawberries, raspberries, other berries, kiwi fruit (one fruit or handful) |
|  | Smoothies (cup) |
|  | Pure fruit juice (100%) e.g. orange, apple juice (cup) |
|  | Grapefruit (half) |
|  | Oranges, satsumas, mandarins (1 fruit) |
|  | Apples (1 fruit) |
|  | Bananas (1fruit) |
|  | Dried fruit, e.g. raisins, prunes (heaped tablespoon) |
|  | Grapes (handful) |
|  | Melon (1 slice) |
|  | Peaches, plums, apricots (1 fruit) |
|  | Pears (1 fruit) |
|  | Tinned fruit (handful) |
| **Whole grains** | |
|  | High Fibre cereals e.g. Branflakes, All Bran, Fruit and Fibre |
|  | Muesli |
|  | Porridge, Readybreak, oats |
|  | Brown rice |
|  | Wholemeal & granary bread/rolls |
|  | Wholemeal pasta |
|  | Crispbread, e.g. Ryvita |
| **Refined grains** | |
|  | Breakfast cereal e.g. Cornflakes, Rice Krispies |
|  | Sugar topped cereals e.g. Frosties |
|  | Naan, poppadoms, flour tortillas |
|  | Brown bread/rolls |
|  | White bread/rolls |
|  | White or green pasta, e.g. spaghetti, macaroni, noodles |
|  | White rice |
| **Nuts and legumes** | |
|  | Beansprouts |
|  | Pulses e.g. lentils, beans, peas |
|  | Green beans, broad beans, runner beans |
|  | Peas |
|  | Baked beans |
|  | Salted nuts e.g. peanuts, cashews (handful) |
|  | Unsalted nuts, e.g. brazil, walnuts (handful) |
|  | Seeds e.g. Sunflower, pumpkin (tablespoon) |
|  | Peanut butter (teaspoon) |
|  | Meat substitutes e.g. tofu, soyameat, textured vegetable protein, vegeburger |
| **Seafood** | |
|  | Oily fish, fresh or canned, e.g. tuna, mackerel, kippers, salmon, sardines, herring |
|  | Fish roe, taramasalata |
|  | Shellfish, e.g. crab, prawns, mussels |
|  | Other white fish, fresh or frozen, e.g. cod, plaice, sole, haddock, halibut |
| **White meat** | |
|  | Chicken or other poultry e.g. turkey |
| **Red, processed meat and eggs** | |
|  | Beef: roast, steak, mince, stew or casserole |
|  | Lamb: roast, chops or stew |
|  | Pork: roast, chops or stew |
|  | Beefburgers |
|  | Bacon or gammon |
|  | Corned Beef, Spam, luncheon meats |
|  | Ham, cured meats & chorizo |
|  | Liver, liver pate, liver sausage |
|  | Sausages |
|  | Eggs as boiled, fried, scrambled, etc. (one) |
| **Fermented dairy** | |
|  | Low fat cheese e.g. reduced fat cheddar (matchbox size) |
|  | Cheese, e.g. cheddar, brie, edam (matchbox size) |
|  | Cottage cheese, low fat soft cheese (2 tablespoons) |
|  | Full fat or Greek yoghurt (small pot) |
|  | Low fat yoghurt, fromage frais (small pot) |
| **Fried foods** | |
|  | Fish fingers, fish cakes & breaded fish |
|  | Fried fish in batter, as in fish and chips |
|  | Chips, retail, fried in vegetable oil |
|  | Potato salad |
|  | Old potatoes, roast in blended oil |
|  | Savoury pies, e.g. meat pie, pork pie, pasties, steak & kidney pie, sausage rolls |
|  | Cream crackers, savoury biscuits |
|  | Crisps or other packet snacks, e.g. Wotsits (one packet) |
|  | Pizza (one slice) |
|  | Quiche (slice) |
| **Sweets and sweet baked products** | |
|  | Reduced fat biscuits e.g. Go Ahead, Highlights (one small packet or one small bar/biscuit) |
|  | Sweet biscuits, chocolate, e.g. digestive (one) |
|  | Sweet biscuits, plain, e.g. Nice, ginger (one) |
|  | Buns, pastries e.g. scones, flapjacks, croissants, doughnuts, home baked |
|  | Cakes e.g. fruit, sponge, home baked |
|  | Cakes e.g. fruit, sponge, ready made |
|  | Fruit pies, tarts, crumbles, home baked |
|  | Fruit pies, tarts, crumbles, ready made |
|  | Milk puddings e.g. rice, custard, trifle |
|  | Sponge puddings, home baked |
|  | Sponge puddings, ready made |
|  | Dairy desserts (small pot) e.g. chocolate mousse, cream caramels |
|  | Ice cream, choc ices |
|  | Jam, marmalade, honey (teaspoon) |
|  | Sugar added to tea, coffee, cereal (teaspoon) |
|  | Sweets, toffees, mints (small packet) |
| **Chocolate** | |
|  | Dark chocolates, single or squares (one) |
|  | White or milk chocolates, single or squares (one) |
|  | Low fat hot chocolate (cup) |
|  | Cocoa, hot chocolate (cup) |
|  | Chocolate snack bars e.g. Mars, Crunchie (one) |
| **Butter and cream** | |
|  | Reduced fat butter (teaspoon) |
|  | Butter (teaspoon) |
|  | Double or clotted cream (tablespoon) |
|  | Single or sour cream (tablespoon) |
| **Spreads and dressings** | |
|  | Low fat spread, e.g. Outline, Gold (teaspoon) |
|  | Very low fat spread (teaspoon) e.g. Diet Flora |
|  | Cholesterol lowering fat spreads e.g. Benecol (teaspoon) |
|  | Olive oil spread (teaspoon) |
|  | Block margarine, e.g. Stork, Krona (teaspoon) |
|  | Other soft margarine, dairy spreads, e.g. Blue Band, Clover (teaspoon) |
|  | Polyunsaturated margarine, e.g. Flora, sunflower (teaspoon) |
|  | French dressing (tablespoon) |
|  | Full fat salad cream, mayonnaise (tablespoon) |
|  | Other salad dressing (tablespoon) |
|  | Low calorie, low fat salad cream (tablespoon) |
| **Milk** | |
|  | Channel Islands milk |
|  | Full cream milk |
|  | Dried milk |
|  | Semi-skimmed milk |
|  | Skimmed milk |
| **Soya and other milk** | |
|  | Goats' milk |
|  | Rice milk |
|  | Soya milk |
| **Soda** | |
|  | Fizzy soft drinks, e.g. Coca Cola, lemonade (cup) |
|  | Low calorie or diet fizzy soft drinks (cup) |
| **Tea** | |
|  | Tea (cup) |
|  | Green tea (cup) |
| **Coffee** | |
|  | Coffee, instant or ground (cup) |
|  | Coffee, decaffeinated (cup) |
| **Alcohol** | |
|  | Beer, lager or cider (half pint) |
|  | Port, sherry, vermouth, liqueurs (pub measure) |
|  | Spirits, e.g. gin, brandy, whisky, vodka (pub measure) |
|  | Red wine (small glass) |
|  | White wine (small glass) |

**Supplementary Text S1: Heritability analysis of the VFM diet score**

Heritability of the VFM diet score was determined using linear structural equation modeling in Mx (Neale & Cardon, 1992; Neale et al., 2003). For univariate ACE modelling the phenotypic variance is decomposed into the additive genetic (A), common environmental (C), and non-shared environmental (E) effect components. When MZ twins are significantly more similar in a phenotype than DZ twins, additive genetic effects are suggested to be high. The influence of family environment is estimated by the common environmental component. The model assumes this is equal in both MZ and DZ twin pairs (Kyvik, 2000). The non-shared environmental component estimates the effects that apply to each individual and includes the measurement error. Heritability is the proportion of the phenotypic variance assigned to genetic factors and is represented by the equation, *h^2^* = (A)/(A + C + E). The ACE, AE, CE, and E models were each tested and the best-fitting model evaluated by the Akaike’s information criterion (AIC). The best model was indicated by the lowest AIC, which reflects the best balance between goodness of fit and parsimony (Neale & Cardon, 1992). Prior to modelling, the VFM diet score was residual-adjusted for age and sex.

References

Kyvik, K. (2000). Generalisability and assumptions of twin studies. In T. D. Spector, H. Snieder & A. J. MacGregor (Eds.), Advances in twin and sib-pair analysis. (pp. 67–77). London: Greenwich Medical Media.

Neale, M. C., Boker, S. M., Xie, G., & Maes, H. (2003). Mx: Statistical modeling. Richmond: Department of Psychiatry, Medical College of Virginia.

Neale, M. C., & Cardon, L. R. (Eds.). (1992). Methodology for genetic studies of twins and families (Nato ISI Series D: Behavioural and Social Sciences, vol. 67). Dordrecht: Kluwer Academic Publishers

.

**Supplementary Text S2: VFM diet score and VFM associations with metabolomics and microbiome**

***VFM diet score and metabolomics and microbiome***

For each metabolite or microbiome taxon, random intercept linear regression analysis was undertaken adjusting for age, BMI, sex and metabolite batch (for metabolomics) or Shannon Index (for microbiome) as fixed effects and family relatedness as a random effect:

$$\Upsilon_{i}=\beta_{0}+\boldsymbol{\beta}_{\boldsymbol{i}}\boldsymbol{X}_{\boldsymbol{ij}}+{\delta_{1i}age}_{ij}+\delta_{2i}{BMI}_{ij}+\delta_{3i}{Sex}_{ij}+\gamma_{1i}Z_{ij}+\zeta_{j}+\varepsilon_{ij}$$

where *Y_i_* is the metabolite/microbiome taxon, *X_ij_* is the VFM diet score of twin *j* from pair *i*, Z_ij_ is the metabolite batch for metabolomics data or Shannon Index for microbiome data and *ζ_j_*, is the family-specific error component that captures the unobserved heterogeneity or family characteristics. We adjusted for multiple testing using Bonferroni correction thus giving a significant threshold of 1.71x10^-4^ (0.05/(292 known metabolites)) for metabolites. Statistical significance assignment for the microbiome was determined within each taxonomic level; details are in **Supplementary Table S2**.

We next conducted a backwards stepwise linear regressions on the significant metabolites to identify a panel of metabolites that were independently associated with the VFM diet score using a cut-off threshold of *P*<0.01. We did not repeat this analysis for the microbiome results as appropriate data reduction procedures are not well established at this time.

***VFM and metabolomics and microbiome***

We used those remaining metabolites, and taxa (residual-adjusted for BMI closest to blood sample) associated with the VFM diet score as predictors of VFM (residual-adjusted for BMI at scan) in a linear regression including scan age, sex, total fat mass, height^2^, scan batch and metabolite batch (for metabolomics) or Shannon Index (for microbiome) as fixed effects and family relatedness as a random effect. The VFM diet score was also added as covariate.

$${VFM}_{i}=\beta_{0}+\boldsymbol{\beta}_{\boldsymbol{1}\boldsymbol{i}}\boldsymbol{Y}\boldsymbol{i} {+ \beta}_{2i}X_{ij}+\delta_{1i}{age}_{ij}+\delta_{3i}{Sex}_{ij}{+ \delta}_{4i}{Total\_fat\_mass}_{ij}+\delta_{5i}{Height\_squared}_{ij}+{\delta_{6i}{Scan\_batch}_{ij}+ \gamma}_{1i}Z_{ij}+\zeta_{j}+\varepsilon_{ij}$$

where *Y_i_* is the metabolite/taxon, *X_ij_* is the VFM diet score of twin *j* from pair *i*, Z_ij_ is the metabolite batch for metabolomics data or Shannon Index for microbiome data and *ζ_j_*, is the family-specific error component that captures the unobserved heterogeneity or family characteristics. Associations were considered significant if they passed the Bonferroni cut-off *P*<5.56x10^-3^ (0.05/9 metabolites) for metabolites, statistical significance thresholds for the microbiome are available in **Supplementary Table S2**.

In MZ twins discordant (1 SD apart) for VFM, a linear regression was run to confirm top associations between diet, metabolites and the microbiome and VFM (metabolomic dataset: *n*=80 pairs; microbiome subsample: *n*=27 pairs).

***Microbiome and metabolite food group intake associations***

To ensure associations between the metabolites/microbiome and the VFM diet score were not driven by reported intakes of other foods the VFM diet score and the food groups not included in the score were fitted into a backward stepwise linear regression model to predict each significant metabolite, taxa and OTU using a cut off threshold of *P*<0.01 for metabolites and *P*<0.05 for microbiome.

To determine if the foods forming the VFM diet score were independently driving the association between the VFM diet score and taxa/metabolites the 20 food groups were fitted into a backward stepwise linear regression model using a cut-off threshold of *P*<0.01 for metabolites and *P*<0.05 for microbiome.

***VFM diet score association with VFM mediated by metabolite and microbiome***

The proportion of the variance of VFM was determined for the VFM diet score after taking into account all covariates (age, sex, BMI, total fat, height^2^, family relatedness, metabolite batch, Shannon Index and scan batch). This quantity is indicated as r^2^_x_. The proportion of the variance for VFM explained by the VFM diet score was then calculated after taking into account the same covariates as above but also including the metabolite or taxon (r^2^_xy_). The percentage of the VFM diet score association mediated by the metabolite or taxon (r^2^_y_.) was calculated as the proportion of the variance of VFM that is due to the VFM diet score association with the metabolite or taxon, namely 1 – (r^2^_xy_/r^2^_x_).

***Microbiome association with VFM mediated by metabolite***

The proportion of the variance of VFM was determined for the taxon after taking into account all covariates as above and the VFM diet score ( r^2^_x_). The proportion of the variance for VFM explained by the taxon was then calculated after taking into account the same covariates as above but also including the metabolite (r^2^_xy_). The percentage of the taxon association mediated by the metabolite (r^2^_y_.) was calculated as the proportion of the variance of VFM that is due to the metabolite association with the taxon, namely 1 – (r^2^_xy_/r^2^_x_).

**Supplementary Table S2. Statistical significance thresholds for microbiome analysis using Bonferroni correction ^(1)^**

|  | **VFM diet score** | |  | **VFM** | |
| --- | --- | --- | --- | --- | --- |
| **Level** | **Number of variables** | ***P*** |  | **Number of variables** | ***P*** |
| Family | 124 | 4.03x10^-4^ |  | 2 | 0.025 |
| Genus | 283 | 1.77x10^-4^ |  | 2 | 0.025 |
| Species | 153 | 3.27x10^-4^ |  | 2 | 0.025 |
| OTU | 2118 | 2.36x10^-5^ |  | 8 | 6.25x10^-3^ |

^(1)^ Bonferroni correction was calculated within each level.

**Supplementary Table S3: Study population characteristics.^1^**

|  |  | **Whole**  **(n=2218)** |  | **Training**  **(n=1109)** |  | **Test**  **(n=1109)** |
| --- | --- | --- | --- | --- | --- | --- |
|  |  | **Mean (SD)** |  | **Mean (SD)** |  | **Mean (SD)** |
| Age (years) |  | 58.3 (10.9) |  | 58.1 (11.1) |  | 58.4 (10.8) |
| BMI (kg/m^2^) |  | 26.2 (4.8) |  | 26.1 (4.8) |  | 26.4 (4.8) |
| Total fat (grams) |  | 6827 (2180) |  | 6779 (2178) |  | 6911 (2258) |
| Visceral fat (grams) |  | 562.7 (293.7) |  | 553.9 (293.8) |  | 572.7 (295.5) |
| Height (m) |  | 1.61 (0.06) |  | 1.62 (0.06) |  | 1.62 (0.06) |
| Sex (M:F) |  | 4:2214 |  | 0:1109 |  | 4:1105 |
| Food intakes (servings/week) |  |  |  |  |  |  |
| Vegetables |  | 34.3 (15.4) |  | 33.8 (15.0) |  | 34.8 (15.8) |
| Fruit and fruit juices |  | 22.4 (13.3) |  | 22.7 (13.8) |  | 22.1 (12.8) |
| Nuts and legumes |  | 7.9 (5.5) |  | 7.8 (5.6) |  | 8.0 (5.4) |
| Wholegrains |  | 10.2 (7.9) |  | 10.2 (7.7) |  | 10.2 (8.1) |
| Refined grains |  | 8.4 (7.4) |  | 8.4 (7.4) |  | 8.5 (7.4) |
| Fermented dairy |  | 6.3 (4.8) |  | 6.4 (4.7) |  | 6.3 (4.8) |
| White meat |  | 1.9 (1.3) |  | 1.9 (1.3) |  | 2.0 (1.3) |
| Seafood |  | 2.4 (2.1) |  | 2.4 (1.9) |  | 2.5 (2.2) |
| Red, processed meat and eggs |  | 6.9 (4.0) |  | 6.9 (3.9) |  | 6.9 (4.0) |
| Fried and fast foods |  | 4.5 (3.4) |  | 4.5 (3.4) |  | 4.6 (3.4) |
| Sweets and sweet baked products |  | 15.7 (13.6) |  | 16.2 (13.3) |  | 15.2 (13.8) |
| Chocolate |  | 4.0 (5.8) |  | 4.1 (6.0) |  | 3.9 (5.7) |
| Butter and cream |  | 4.4 (6.6) |  | 4.7 (6.8) |  | 4.1 (6.4) |
| Spreads and dressings |  | 8.3 (8.4) |  | 8.4 (8.8) |  | 8.2 (8.1) |
| Soy and other milks |  | 0.2 (0.8) |  | 0.2 (0.8) |  | 0.2 (0.8) |
| Milk |  | 3.9 (4.2) |  | 3.8 (4.1) |  | 4.0 (4.3) |
| Soda |  | 2.0 (5.2) |  | 1.9 (5.5) |  | 2.1 (4.9) |
| Coffee |  | 8.7 (10.4) |  | 8.8 (10.5) |  | 8.6 (10.4) |
| Tea |  | 19.3 (13.7) |  | 19.5 (13.8) |  | 19.1 (13.6) |
| Alcohol |  | 6.0 (8.0) |  | 5.7 (7.8) |  | 6.3 (8.3) |

(1) No significant differences between the training and testing sets.

**Supplementary Table S4: Results of the structural equation modeling for heritability of the visceral fat mass diet score**

| **Model** |  | **A** |  | **C** |  | **E** | **log-likelihood** | **Chi-squared** | **df** | ***P*** | **AIC** |
| --- | --- | --- | --- | --- | --- | --- | --- | --- | --- | --- | --- |
| ***Saturated*** |  | 0.44 (0.31, 0.50) |  | 0.00 (0.00, 0.11) |  | 0.56 (0.50, 0.63) | 9067 |  | 1787 |  | 5493 |
| ***AE**** |  | 0.44 (0.37, 0.50) |  | - |  | 0.56 (0.50, 0.63) | 9067 | 0.00 | 1788 | Incalc | 5491 |
| ***CE*** |  | - |  | 0.33 (0.27, 0.38) |  | 0.67 (0.62, 0.73) | 9090 | 23.40 | 1788 | 0.000 | 5514 |
| ***E*** |  | - |  | - |  | 1.00 (1.00, 1.00) | 9192 | 124.70 | 1789 | 0.000 | 5613 |

*Best fitting model

**Supplementary Table S5: Nutrient profile and linear trends of the visceral fat mass diet score according to score tertile ^(1)^**

|  | Tertile 1 | Tertile 2 | Tertile 3 | Trend | |  | Tertile 1 | Tertile 2 | Tertile 3 | Trend | |
| --- | --- | --- | --- | --- | --- | --- | --- | --- | --- | --- | --- |
| Nutrient | Mean (SD) | Mean (SD) | Mean (SD) | Beta (SE) | *P* | Nutrient | Mean (SD) | Mean (SD) | Mean (SD) | Beta (SE) | *P* |
| Energy (kcal) | 1892.1 (510.9) | 1804.2 (522.9) | 1870.4 (552.2) | -15.61 (14.56) | NS | Calcium (mg/d) | 1095.7 (286.9) | 1039.4 (260.8) | 949.3 (264.1) | -72.17 (7.31) | 3.18x10^-22^ |
| Fat (g/d) | 61.5 (10.4) | 68.6 (9.5) | 72.4 (10.3) | 5.53 (0.28) | 1.62x10^-75^ | Magnesium (mg/d) | 369.1 (52.3) | 336.3 (49.2) | 296.7 (42.4) | -36.01 (1.27) | 3.72x10^-138^ |
| Saturated FAs (g/d) | 21.3 (5.4) | 24.5 (5.2) | 26.6 (5.1) | 2.65 (0.14) | 1.90x10^-67^ | Phosphorous (mg/d) | 1534.9 (218.8) | 1474.9 (202.7) | 1357 (211.8) | -87.14 (5.81) | 4.40x10^-47^ |
| MUFAs (g/d) | 19.7 (3.7) | 22.7 (3.4) | 24.7 (3.8) | 2.54 (0.1) | 2.33x10^-113^ | Iron (mg/d) | 13.5 (3.1) | 12.5 (2.4) | 11.5 (2.3) | -0.99 (0.07) | 2.43x10^-39^ |
| PUFAs (g/d) | 15.2 (4.4) | 15.9 (4.4) | 15.7 (4.5) | 0.3 (0.12) | 1.21x10^-2^ | Copper (mg/d) | 1.6 (0.4) | 1.6 (0.5) | 1.5 (0.5) | -0.07 (0.01) | 1.90x10^-8^ |
| trans-FAs (g/d) | 1.3 (0.6) | 1.6 (0.6) | 1.8 (0.6) | 0.25 (0.02) | 2.55x10^-51^ | Zinc (mg/d) | 10.1 (1.5) | 10.1 (1.6) | 9.8 (1.7) | -0.13 (0.04) | 2.60x10^-3^ |
| Cholesterol (mg/d) | 202.5 (69.1) | 234.8 (74.2) | 251.5 (78.2) | 24.98 (2.06) | 4.04x10^-32^ | Manganese (mg/d) | 4.4 (1.1) | 4 (1) | 3.6 (0.9) | -0.43 (0.03) | 9.54x10^-52^ |
| Protein (g/d) | 79.5 (12.2) | 81 (12.3) | 79.6 (12.5) | 0.15 (0.34) | NS | Iodine (ug/d) | 212.9 (73) | 207 (68.7) | 196.5 (68.7) | -8.03 (1.89) | 2.25x10^-5^ |
| Carbohydrate (g/d) | 248.1 (29.6) | 229 (29.1) | 217.2 (31.1) | -15.66 (0.84) | 1.13x10^-69^ | Retinol (ug/d) | 407.1 (403.2) | 533.9 (586.6) | 617.4 (772.6) | 106.49 (16.78) | 3.05x10^-10^ |
| Starch (g/d) | 107.7 (26.3) | 107.9 (25.1) | 111.2 (26.1) | 1.65 (0.71) | 2.05x10^-2^ | Carotene (ug/d) | 6060.1 (4193.3) | 5399.6 (3002.5) | 4729.8 (2742.7) | -664.87 (94.64) | 3.41x10^-12^ |
| Total sugars (g/d) | 137.5 (28.1) | 118.3 (24.4) | 103.4 (24.1) | -17.22 (0.71) | 5.34x10^-108^ | Vitamin D (ug/d) | 2.5 (1.1) | 2.5 (1) | 2.4 (0.9) | -0.05 (0.03) | 5.36x10^-2^ |
| Glucose (g/d) | 28 (9.2) | 22.4 (7.8) | 18 (6.5) | -5.05 (0.22) | 8.20x10^-101^ | Vitamin E (mg/d) | 11.7 (3.4) | 10.9 (2.9) | 9.9 (2.8) | -0.92 (0.08) | 1.02x10^-26^ |
| Fructose (g/d) | 33.7 (11.4) | 26 (9.2) | 19.9 (7.5) | -6.99 (0.26) | 1.29x10^-125^ | Thiamin (mg/d) | 1.8 (0.4) | 1.7 (0.3) | 1.6 (0.3) | -0.12 (0.01) | 3.38x10^-31^ |
| Sucrose (g/d) | 48 (14.1) | 44.2 (14.4) | 42.7 (15.6) | -2.73 (0.41) | 6.34x10^-11^ | Riboflavin (mg/d) | 2.3 (0.7) | 2.2 (0.6) | 2.1 (0.6) | -0.09 (0.02) | 2.03x10^-7^ |
| Maltose (g/d) | 3.3 (1.5) | 3 (1.6) | 3.3 (1.8) | -0.02 (0.05) | NS | Niacin (mg/d) | 21.7 (5) | 21.1 (4.5) | 20.5 (4.5) | -0.59 (0.13) | 4.09x10^-6^ |
| Lactose (g/d) | 18.7 (9.9) | 18.3 (8.9) | 17 (9.8) | -0.82 (0.26) | 1.82x10^-3^ | Tryptophan (mg/d) | 16.6 (2.5) | 16.9 (2.5) | 16.6 (2.5) | 0.02 (0.07) | NS |
| NSP (g/d) | 22.9 (5.5) | 19.6 (4.5) | 16.4 (4.2) | -3.22 (0.13) | 2.27x10^-110^ | Vitamin B6 (mg/d) | 2.6 (0.6) | 2.5 (0.5) | 2.3 (0.5) | -0.15 (0.01) | 2.63x10^-23^ |
| Alcohol (g/d) | 8.4 (11.1) | 8.8 (11.3) | 10.9 (14.9) | 1.24 (0.38) | 9.45x10^-4^ | Vitamin B12 (mg/d) | 5.8 (2.2) | 6.3 (2.5) | 6.4 (2.9) | 0.31 (0.07) | 6.65x10^-6^ |
| Water (g/d) | 2754.6 (662) | 2593.7 (598.9) | 2478.5 (598.1) | -139.45 (16.71) | 1.80x10^-16^ | Folate (ug/d) | 416 (116) | 378.8 (103.5) | 341.8 (91.7) | -37.1 (2.79) | 5.95x10^-38^ |
| Sodium (mg/d) | 2308.6 (498.6) | 2262.8 (471.7) | 2162.2 (453.5) | -71.5 (12.87) | 3.33x10^-8^ | Pantothenate (mg/d) | 6.1 (1.6) | 6 (2.8) | 5.6 (1.3) | -0.23 (0.04) | 1.43x10^-8^ |
| Potassium (mg/d) | 4080.4 (624.4) | 3833 (569.2) | 3550.3 (524.2) | -263.97 (15.56) | 1.48x10^-58^ | Biotin (ug/d) | 46.9 (10.1) | 45.5 (9.4) | 42.3 (9.5) | -2.27 (0.26) | 1.50x10^-17^ |
| Chloride (mg/d) | 3705.3 (764.9) | 3598.8 (728.6) | 3411.9 (694.5) | -144.23 (19.7) | 4.30x10^-13^ | Vitamin C (mg/d) | 198.9 (81.5) | 164.1 (66.9) | 125.8 (50.8) | -36.44 (1.82) | 8.20x10^-78^ |

NS= not significant: P>0.05. FA: fatty acid, MUFAS: monounsaturated fatty acids, PUFAS: polyunsaturated fatty acids, NSP: non-starch polysaccharides

1. Tertile 1: score 0-6; tertile 2: score 7-9; tertile 3: scores 10-15. Linear trend determined by using the tertile of the VFM diet score as a predictor of the energy-adjusted nutrient intake.

**Supplementary Table S6: List of metabolites significantly associated with the VFM diet score and with each food group independently (*P*<0.01 backward stepwise regression).**

|  |  | VFM Score ^(1)^ | | VFM Score adjusted foods ^(2)^ | | Foods associated ^(3)^ | |
| --- | --- | --- | --- | --- | --- | --- | --- |
| Metabolite | **Sub-pathway** | **Beta (SE)** | ***P*** | **Beta (SE)** | ***P*** | ***P*<0.01** | ***P*<3.33x10^-4^** |
| Eicosapentaenoate | Essential fatty acid | -0.058(0.007) | 3.24x10^-17^ | -0.052(0.006) | 2.22x10^-15^ | Fruit (0.005(0.002))  FD (0.011(0.004))  FF (-0.017(0.006)) | WG (0.011(0.003)) |
| Indolepropionate | Tryptophan metabolism | -0.056(0.007) | 1.58x10^-16^ | -0.054(0.007) | 2.91x10^-16^ | Fruit (0.006(0.002))  WG (0.008(0.003))  RM (-0.018(0.005)) |  |
| 3-Carboxy-4-methyl-5-propyl-2-furanpropanoate | Fatty acid, dicarboxylate | -0.055(0.007) | 2.43x10^-16^ | -0.045(0.006) | 3.19x10^-12^ | FF (-0.023(0.007)) | WG (0.013(0.003)) |
| Docosahexaenoate | Essential fatty acid | -0.054(0.007) | 6.17x10^-16^ | -0.045(0.006) | 1.30x10^-12^ | WG (0.010(0.003)) | Fruit (0.008(0.002)) |
| Stachydrine | Food component, Plant | -0.056(0.007) | 7.95x10^-16^ | -0.057(0.007) | 2.59x10^-16^ | RM (-0.019(0.006)) | Fruit (0.014(0.002)) |
| 3-Phenylpropionate | Phenylalanine & tyrosine metabolism | -0.059(0.007) | 1.70x10^-15^ | -0.053(0.008) | 3.53x10^-12^ | Fruit (0.006(0.002)) | WG (0.014(0.003))  FF (-0.028(0.007)) |
| Hippurate | Benzoate metabolism | -0.052(0.007) | 1.84x10^-13^ | -0.052(0.007) | 5.06x10^-14^ |  | Fruit (0.010(0.002))  WG (0.011(0.002)) |
| Catechol sulfate | Benzoate metabolism | -0.051(0.007) | 1.75x10^-12^ | -0.046(0.007) | 1.94x10^-10^ | WG (0.008(0.003)) | Fruit (0.008(0.002)) |
| Glycerate | Glycolysis, gluconeogenesis, pyruvate metabolism | -0.049(0.007) | 3.81x10^-12^ | -0.042(0.007) | 1.57x10^-9^ |  | Fruit (0.010(0.002)) |
| Pyridoxate | Vitamin B6 metabolism | -0.049(0.007) | 9.08x10^-12^ | -0.042(0.007) | 2.96x10^-9^ |  | Fruit (0.007(0.002))  WG (0.011(0.002)) |
| Threitol | Nucleotide sugars, pentose metabolism | -0.044(0.007) | 7.73x10^-11^ | -0.043(0.007) | 1.65x10^-10^ |  | Fruit (0.011(0.002)) |
| Butyrylcarnitine | Fatty acid metabolism (also BCAA metabolism) | 0.040(0.007) | 4.77x10^-9^ | 0.039(0.007) | 9.09x10^-9^ |  | RM (0.020(0.005)) |
| alpha-Hydroxyisovalerate | Valine, leucine and isoleucine metabolism | 0.037(0.007) | 1.12x10^-7^ | 0.027(0.006) | 4.48x10^-5^ |  | RM (0.022(0.005)) |
| 1-Arachidonoylglycerophosphoethanolamine* | Lysolipid | 0.038(0.007) | 2.09x10^-7^ | 0.030(0.007) | 3.43x10^-5^ | WG (-0.011(0.003))  FD (-0.013(0.005)) |  |
| Threonate | Ascorbate and aldarate metabolism | -0.036(0.007) | 4.51x10^-7^ | -0.033(0.007) | 3.66x10^-6^ |  | Fruit (0.007(0.002)) |
| 1,5-Anhydroglucitol (1,5-AG) | Glycolysis, gluconeogenesis, pyruvate metabolism | 0.035(0.007) | 1.80x10^-6^ | 0.026(0.007) | 3.46x10^-4^ |  | Fruit (-0.008(0.002)) |
| Uridine | Pyrimidine metabolism, uracil containing | -0.032(0.007) | 2.05x10^-6^ | -0.03(0.007) | 9.21x10^-6^ | WG (0.008(0.003)) |  |
| Stearidonate | Long chain fatty acid | -0.033(0.007) | 2.65x10^-6^ | -0.028(0.007) | 3.20x10^-5^ | None |  |
| 1-Docosahexaenoylglycerophosphocholine* | Lysolipid | -0.032(0.007) | 2.95x10^-6^ | -0.027(0.007) | 4.14x10^-5^ | FD (0.014(0.004)) |  |
| Bilirubin (Z,Z) | Hemoglobin and porphyrin metabolism | -0.033(0.007) | 3.88x10^-6^ | -0.032(0.007) | 4.69x10^-6^ |  | FF (-0.020(0.006)) |
| 1-Oleoylglycerophosphoethanolamine | Lysolipid | 0.032(0.007) | 9.21x10^-6^ | 0.023(0.007) | 1.35x10^-3^ | WG (-0.008(0.003)) |  |
| 1-Arachidonoylglycerophosphocholine* | Lysolipid | 0.032(0.007) | 1.76x10^-5^ | 0.024(0.007) | 1.26x10^-3^ | WG (-0.010(0.003)) |  |
| Pantothenate | Pantothenate and CoA metabolism | -0.029(0.007) | 4.20x10^-5^ | -0.029(0.007) | 6.28x10^-5^ | None |  |
| 4-Androsten-3beta,17beta-diol disulfate 1* | Sterol, Steroid | 0.032(0.008) | 4.67x10^-5^ | 0.019(0.007) | 7.86x10^-3^ | None |  |
| X-11793--oxidized bilirubin* | Hemoglobin and porphyrin metabolism | 0.028(0.007) | 5.10x10^-5^ | 0.027(0.007) | 7.01x10^-5^ | WG (-0.009(0.002))  FD (-0.015(0.005)) |  |
| trans-4-Hydroxyproline | Urea cycle; arginine-, proline-, metabolism | 0.027(0.007) | 8.28x10^-5^ | 0.031(0.007) | 7.13x10^-6^ | FD (-0.013(0.005)) | RM (0.028(0.006)) |
| Glycoursodeoxycholate | Bile acid metabolism | 0.032(0.008) | 1.00x10^-4^ | 0.028(0.008) | 5.70x10^-4^ | None |  |
| gamma-Glutamylvaline | gamma-glutamyl | 0.023(0.006) | 1.11x10^-4^ | 0.023(0.006) | 1.38x10^-4^ | FD (-0.014(0.004)) | Fruit (-0.005(0.001)) |
| 1-Eicosatrienoylglycerophosphocholine* | Lysolipid | 0.028(0.007) | 1.32x10^-4^ | 0.020(0.007) | 4.40x10^-3^ | WG (-0.009(0.003)) |  |
| Proline | Urea cycle; arginine-, proline-, metabolism | 0.028(0.007) | 1.44x10^-4^ | 0.028(0.007) | 1.45x10^-4^ | FF (0.015(0.006)) | Fruit (-0.008(0.002)) |

FD: Fermented dairy; FF: Fried and fast foods; RM: Red meat; WG: Wholegrain products

1. Metabolite associations with the VFM diet score were adjusted for covariates (batch effects, age, BMI and sex) and multiple testing (P<1.71x10^-4^).
2. The VFM diet score and 15 food groups not forming the score were fitted into a backward stepwise linear regression model to predict each significant metabolite using P<0.01 as the cut off threshold.
3. All 20 food groups were fitted into a backward stepwise linear regression model to predict each significant metabolite using P<0.01 as the cut off threshold. Significant results shown only for foods forming the VFM diet score. Associations passing the Bonferonni cut-off were considered statistically significant: *P*<3.33x10^-4^ = 0.05/[5 food groups x 30 metabolites].

|  |  |  |  |  | Regression | | |
| --- | --- | --- | --- | --- | --- | --- | --- |
|  |  | Low-VFM twins | High-VFM twins |  | VFM | | |
| Variable | N pairs | Mean(SE) | Mean(SE) |  | Beta(SE) | *P* | R^2^ |
| VFM diet score | 80 | -0.141(0.122) | -0.047(0.115) |  | 0.281(0.091) | 0.002 | 0.057 |
| Alpha-hydroxyisovalerate | 80 | -0.239(0.120) | 0.064(0.111) |  | 0.141(0.094) | 0.136 | 0.014 |
| Bilirubin (Z,Z) | 68 | 0.093(0.102) | -0.110(0.134) |  | -0.187(0.102) | 0.068 | 0.023 |
| Butyrylcarnitine | 80 | -0.168(0.126) | 0.096(0.124) |  | 0.199(0.087) | 0.023 | 0.032 |
| Hippurate | 80 | 0.062(0.112) | -0.162(0.114) |  | -0.297(0.095) | 0.002 | 0.058 |
| Shannon Index | 27 | -0.019(0.182) | -0.268(0.224) |  | -0.287(0.176) | 0.110 | 0.048 |
| *Bifidobacterium* OTU (4426298) | 27 | 0.140(0.718) | 0.175(1.098) |  | -0.064(0.197) | 0.749 | 0.002 |
| *Eubacterium dolichum* | 27 | -0.108(0.155) | -0.013(0.171) |  | 0.131(0.227) | 0.566 | 0.006 |

**Supplementary Table S7. Associations between visceral fat mass (VFM) and the VFM diet score, Shannon Index and top microbiome and metabolite associations in the MZ discordant twin sample ^(1)^**

1. A linear regression was conducted using the VFM diet score, Shannon Index and top microbiome and metabolite associations to predict VFM in the MZ discordant (1 SD apart in VFM) twin sample. Variables standardized to have mean=0, SD=1.

**Supplementary Table S8. List of OTUs associated with the VFM diet score (unadjusted and adjusted for other food intakes), their association with foods forming the VFM diet score and their independent association with the VFM diet score (*P*<0.05 in backward linear regression).**

|  |  | VFM score ^(1)^ | | VFM score adjusted foods ^(2)^ | | Foods associated ^(3)^ |
| --- | --- | --- | --- | --- | --- | --- |
| OTU ID | **Assigned Taxonomy** | Beta(SE) | *P* | Beta(SE) | *P* | *P*<0.05 |
| 4426298 ^(4)^ | k__Bacteria; p__Actinobacteria; c__Actinobacteria; o__Bifidobacteriales; f__Bifidobacteriaceae; g__Bifidobacterium; s__ | -0.058(0.011) | 6.19x10^-7^ | -0.053(0.011) | 9.56x10^-7^ | FD (0.029(0.008))*  FF (-0.022(0.010)) |
| 183686 | k__Bacteria; p__Firmicutes; c__Clostridia; o__Clostridiales; f__Ruminococcaceae; g__; s__ | 0.053(0.011) | 1.25x10^-6^ | 0.042(0.011) | 9.01x10^-5^ | RM (0.018(0.009))  WG (-0.016(0.004))* |
| 592616 | k__Bacteria; p__Firmicutes; c__Erysipelotrichi; o__Erysipelotrichales; f__Erysipelotrichaceae; g__; s__ | 0.050(0.011) | 3.35x10^-6^ | 0.042(0.010) | 4.48x10^-5^ | RM (0.027(0.009))* |
| 2368865 | k__Bacteria; p__Firmicutes; c__Clostridia; o__Clostridiales; f__; g__; s__ | -0.047(0.011) | 1.11x10^-5^ | -0.044(0.010) | 2.40x10^-5^ | FD (0.031(0.006))* |
| 509709 | k__Bacteria; p__Firmicutes; c__Clostridia; o__Clostridiales; f__Lachnospiraceae; g__; s__ | -0.051(0.011) | 1.15x10^-5^ | -0.047(0.011) | 2.57x10^-5^ | RM (0.011(0.004))  WG (-0.023(0.008)) |
| New.0.ReferenceOTU51 | k__Bacteria; p__Bacteroidetes; c__Bacteroidia; o__Bacteroidales; f__Rikenellaceae; g__; s__ | 0.049(0.011) | 1.44x10^-5^ | 0.045(0.011) | 3.87x10^-5^ | WG (-0.011(0.004)) |
| 3801267 | k__Bacteria; p__Firmicutes; c__Clostridia; o__Clostridiales; f__Veillonellaceae; g__Veillonella; s__parvula | -0.043(0.011) | 1.75x10^-5^ | -0.039(0.010) | 7.29x10^-5^ | Fruit (0.006(0.002)) |
| 2407149 | k__Bacteria; p__Firmicutes; c__Clostridia; o__Clostridiales; f__Lachnospiraceae; g__; s__ | -0.043(0.010) | 2.24x10^-5^ | -0.028(0.010) | 0.006 | None |

*= statistically significant: *P*<0.0025; FD: fermented dairy; FF: fried and fast foods; RM: red meat; WG: wholegrain products

1. OTU associations with the VFM diet score were adjusted for covariates (age, Shannon Index, BMI and sex) and multiple testing (*P*<2.36x10^-5^ [Bonferroni: 0.05/2118 OTUs]).
2. The VFM diet score and 15 food groups not forming the score were fitted into a backward stepwise linear regression model to predict each significant OTU using *P*<0.05 as the cut off threshold.
3. All 20 food groups were fitted into a backward stepwise linear regression model to predict each significant OTU using *P*<0.05 as the cut off threshold. Significant results shown only for foods forming the VFM diet score.
4. OTU 4426298 is the only taxon associated with VFM independently of the VFM diet score (Beta[SE]: (-0.046[0.016]; *P*=0.005)
